# Demographic Clusters Identified within the Northern Gulf of Mexico Common Bottlenose Dolphin (*Tursiops truncates*) Unusual Mortality Event: January 2010 - June 2013

**DOI:** 10.1371/journal.pone.0117248

**Published:** 2015-02-11

**Authors:** Stephanie Venn-Watson, Lance Garrison, Jenny Litz, Erin Fougeres, Blair Mase, Gina Rappucci, Elizabeth Stratton, Ruth Carmichael, Daniel Odell, Delphine Shannon, Steve Shippee, Suzanne Smith, Lydia Staggs, Mandy Tumlin, Heidi Whitehead, Teri Rowles

**Affiliations:** 1 National Marine Mammal Foundation, San Diego, California, United States of America; 2 National Marine Fisheries Service, Southeast Fisheries Science Center, Miami, Florida, United States of America; 3 National Marine Fisheries Service, Southeast Regional Office, St. Petersburg, Florida, United States of America; 4 NOAA Affiliate, Southeast Fisheries Science Center, Miami, Florida, United States of America; 5 Dauphin Island Sea Lab and University of South Alabama, Dauphin Island, Alabama, United States of America; 6 Hubbs-SeaWorld Research Institute, Melbourne Beach, Florida, United States of America; 7 Institute for Marine Mammal Studies, Gulfport, Mississippi, United States of America; 8 Emerald Coast Wildlife Refuge, Fort Walton Beach, Florida, United States of America; 9 Marine Wildlife Response, Esther, Florida, United States of America; 10 Audubon Aquarium of the Americas, New Orleans, Louisiana, United States of America; 11 Gulf World Marine Park, Panama City Beach, Florida, United States of America; 12 Louisiana Department of Wildlife and Fisheries, Baton Rouge, Louisiana, United States of America; 13 Texas Marine Mammal Stranding Network, Galveston, Texas, United States of America; 14 National Marine Fisheries Service, Office of Protected Resources, Silver Spring, Maryland, United States of America; American University in Cairo, EGYPT

## Abstract

A multi-year unusual mortality event (UME) involving primarily common bottlenose dolphins (*Tursiops truncates*) was declared in the northern Gulf of Mexico (GoM) with an initial start date of February 2010 and remains ongoing as of August 2014. To examine potential changing characteristics of the UME over time, we compared the number and demographics of dolphin strandings from January 2010 through June 2013 across the entire GoM as well as against baseline (1990-2009) GoM stranding patterns. Years 2010 and 2011 had the highest annual number of stranded dolphins since Louisiana’s record began, and 2011 was one of the years with the highest strandings for both Mississippi and Alabama. Statewide, annual numbers of stranded dolphins were not elevated for GoM coasts of Florida or Texas during the UME period. Demographic, spatial, and temporal clusters identified within this UME included increased strandings in northern coastal Louisiana and Mississippi (March-May 2010); Barataria Bay, Louisiana (August 2010-December 2011); Mississippi and Alabama (2011, including a high prevalence and number of stranded perinates); and multiple GoM states during early 2013. While the causes of the GoM UME have not been determined, the location and magnitude of dolphin strandings during and the year following the 2010 *Deepwater Horizon* oil spill, including the Barataria Bay cluster from August 2010 to December 2011, overlap in time and space with locations that received heavy and prolonged oiling. There are, however, multiple known causes of previous GoM dolphin UMEs, including brevetoxicosis and dolphin morbillivirus. Additionally, increased dolphin strandings occurred in northern Louisiana and Mississippi before the *Deepwater Horizon* oil spill. Identification of spatial, temporal, and demographic clusters within the UME suggest that this mortality event may involve different contributing factors varying by location, time, and bottlenose dolphin populations that will be better discerned by incorporating diagnostic information, including histopathology.

## Introduction

In the United States, an increase in marine mammal strandings may be defined as an unusual mortality event (UME) based on a legislative process, regardless of etiology or absolute numbers of animals affected [1]. The 1992 amendments to the Marine Mammal Protection Act define a UME as an unexpected event involving a significant die-off of any marine mammal population, and demanding immediate response. Declaration of a UME is based upon comparisons of characteristics of a stranding event with historical data; review and recommendation by a federally appointed Working Group for Marine Mammal Unusual Mortality Events; and input from the National Oceanic and Atmospheric Administration (NOAA) National Marine Fisheries Service (NMFS), the Marine Mammal Commission, and the U.S. Fish and Wildlife Service.

On December 13, 2010, a UME was declared involving cetaceans in the northern Gulf of Mexico (GoM) beginning February 2010 and continuing into 2014 [2]. The UME region has been defined as extending from the Texas/Louisiana border through Franklin County, Florida. High monthly stranding rates were determined by months in which stranded cetacean numbers exceed mean baseline (2002–2009) stranding numbers plus two times the standard deviation [1,2]. Based on documentation of previous UMEs, the ongoing GoM cetacean UME is the longest marine mammal die-off in the stranding record for the GoM [2]. More than 95% of cetaceans have stranded dead, and the causes remain unknown. While the majority (87%) of stranded cetaceans have been common bottlenose dolphins (*Tursiops truncatus*), other cetaceans that stranded during this UME include (but are not limited to) spinner dolphins (*Stenella longirostris*), Atlantic spotted dolphins (*Stenella frontalis*), and melon-headed whales (*Peponocephala electra*) [2].

The ongoing event is not this region’s first UME. Prior to 2010, ten UMEs involving bottlenose dolphins have been documented in the GoM, as well as one die-off that occurred in 1990, prior to the establishment of the UME program [2]. Morbillivirus infections and brevetoxicosis have been the most common known or suspected causes of previous GoM UMEs [2–6]. Brevetoxin-associated UMEs, however, have been limited in distribution (e.g. Florida panhandle), and neither brevetoxins nor morbillivirus have been known to cause die-offs lasting as long as the ongoing GoM UME [2, 4–6]. The unusually long duration and geographic scope of the ongoing GoM UME are suggestive of alternate or multiple contributors compared to previous cetacean die-offs in this region.

Approximately 3 months after the declared start date of the ongoing GoM UME, the *Deepwater Horizon* (DWH) oil spill occurred on April 20, 2010 [7]. By the time the well was permanently sealed on September 19, 2010, an estimated 4.9 million barrels of oil were discharged into the GoM, making it the largest marine oil spill in U.S. history [8]. In the days and months following initial release of oil, there were large increases in mortalities of birds, turtles, and mammals [9]. A combination of factors, including the oil spill and colder than normal sea surface temperatures, was proposed as having contributed to increased dolphin deaths in the northern GoM during 2011 [10].

To assess the potential impact of the DWH oil spill on dolphins, health assessments were conducted on live common bottlenose dolphins living in Barataria Bay, Louisiana, a coastal area heavily impacted by the spill [11]. This assessment revealed a high prevalence of moderate to severe lung disease and evidence of hypoadrenocorticism in Barataria Bay dolphins. These abnormalities were consistent with adverse health effects that might be expected following oil exposure based upon the literature of documented effects in other animal species [11].

Common bottlenose dolphins in the GoM are subdivided into stocks, and dolphins within bay, sound, and estuary stocks appear to have high site fidelity and minimal migration [12]. Almost all of the stranded dolphins from the ongoing UME genotyped to date have been confirmed as the nearshore ecotype [2]. Similar to dolphins in other regions, northern GoM dolphins have a variety of adaptable feeding behaviors based upon their habitats and diversity of available prey [13]. Calving season for dolphins in the northern GoM is typically in the spring, and as such, there is an annual peak of strandings, including an increase in perinatal dolphins, that occur during February to April. [14].

Multiple factors may be contributing across regions and over time to this prolonged UME. As such, there was a need to assess potential spatial, temporal and demographic (age, sex class) clusters of cetacean, particularly common bottlenose dolphin, strandings within the UME while accounting for expected seasonal stranding peaks. Further, to better compare numbers of stranded dolphins during the UME across the entire GoM and to compare this to historical GoM dolphin strandings, there was a need to develop a more robust baseline that involved information from longer term datasets (1990–2010) and adjusted for variations in historical stranding response coverage. Further, due to the heavy oiling and health assessment findings in dolphins in Barataria Bay, Louisiana, there was a need to assess the time and relative magnitude of dolphin strandings in Barataria Bay. Results from this analysis will help with the development of subsequent diagnostic investigations by demographic and geographic stranding clusters.

## Materials and Methods

### Stranding data

Cetacean and pinniped (except walrus) strandings in the U.S. are monitored by the Marine Mammal Health and Stranding Response Program (MMHSRP) within NMFS. Response to cetacean stranding events is conducted largely by volunteer Marine Mammal Stranding Networks (MMSN) authorized under Section 112c (Stranding Agreements from the NMFS regional offices) or Section 109h (Federal, State, or local government officials) of the Marine Mammal Protection Act. A standardized set of demographic data (known as Level A data) are collected on stranded marine mammals and submitted by MMSN responders to regional and national NMFS databases. This study’s data were extracted from the Historical Southeastern U.S. Marine Mammal Stranding Database (1990–1995) and the MMHSRP National Marine Mammal Stranding Database (1996—June 2013). Level A data can be amended if more information becomes available, however, the data used for this study were the most accurate available when extracted in December 2013. Retrieved data for each individual animal included its unique identifier, genus (*Tursiops*), stranding location (state, county, latitude and longitude), sex, total body length, and date of initial observation.

### Definitions

Although the northern GoM UME includes many different cetacean species, the majority (87%) of stranded cetaceans have been common bottlenose dolphins. It was therefore decided to limit this study to data related to common bottlenose dolphins (hereafter referred to as ‘dolphins’) identified as *Tursiops truncatus* either visually or through genetic analyses that stranded on U.S. GoM coastlines (Texas, Louisiana, Mississippi, Alabama, and the Gulf coast of Florida including Monroe County). Stranding location was assigned to the county from which the carcass or animal was recovered. Comparisons of annual stranding data were limited to those strandings reported from January 1990 through December 2012; data before 1990 were not as consistently recorded and were not included in the analysis. We defined baseline years as those prior to the start of the northern GoM cetacean UME (1990–2009). Years and states with previous UMEs were included as part of the baseline to assess how much higher or lower 2010–2012 strandings were relative to a region with a history of numerous UMEs. Total annual data for 2013 were incomplete and not included in the statewide, annual analyses.

Demographics of stranded dolphins were defined in terms of sex (male or female) and age class (perinate or non-perinate). Perinates were defined as dolphins with a reported whole body length less than 115 cm (rostrum to fluke notch) based on reported dolphin lengths at birth, which are 90 to 103 cm for females and 100 to 107 cm for males [14, 15]. Before 2007, body length reported for’whole' or’partial' carcass was not distinguished. Hence, data records before 2007 may overestimate the number of perinates due to incorrect assignment of partial carcasses to this age class. Since it would be difficult to determine which data to exclude for this period, to ensure the highest possible numbers for robust statistical analyses, we opted to include all recorded body lengths, including those prior to 2007.

### Large-scale mortality years

The term, large-scale mortality year (LSMY), was used to define years in which the total number of dolphin strandings were equal to or exceeded the 95^th^ quantile calculated from the baseline years in each state (1990–2009). State-years in which there were no reported dolphin strandings (Louisiana, years 1991–1992 and 2001–2002) were excluded as part of the baseline, since this low number was likely due to poor coverage. For Florida, the analysis was limited to the Gulf coast (“Gulf Florida”) which was defined as all strandings in Monroe County north to the Florida-Alabama state line. Statistical analyses were carried out using SAS Release 9.2 (SAS Incorporated, Cary, North Carolina). Once LSMYs were identified, comparisons of stranding demographics among them were conducted based upon potential similarities of previous LSMYs in size or location to or within the ongoing UME. LSMYs were compared using Chi-square tests for sex (males and females) and age class (perinates and non-perinates). Wilcoxon rank-sum tests were used to compare body lengths in different LSMYs. Significance was defined as a *P* value less than or equal to 0.05.

### Regional monthly baseline stranding numbers

**Fig 1:**
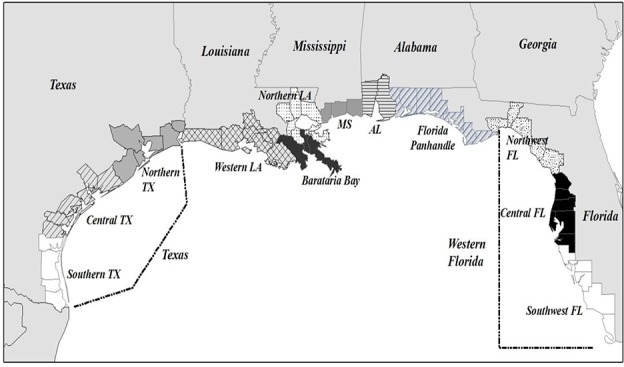
Map of the U.S. Gulf Coast showing county groups used in analysis of regional monthly baseline common bottlenose dolphin (*Tursiops truncatus*) stranding numbers. Baselines for Texas and Western Florida were determined by combining multiple regions outlined below. Gulf Florida includes the Florida Panhandle and the Western Florida combined region.

To define monthly regional stranding baselines, GoM coastal counties were grouped into state boundaries, with the exception of Louisiana and Gulf Florida (Fig. 1). Louisiana was broken into three regions due to its sustained high stranding numbers during the UME. Dolphin strandings from Western Louisiana were defined as dolphins that were observed stranded in Cameron Parish east through Terrebonne Parish. Barataria Bay, Louisiana was defined as Lafourche, Jefferson, and Plaquemines parishes and Northern Louisiana was defined as St. Bernard, Orleans, St. Charles, St. John Baptist, Livingston, Tangipahoa and St. Tammany Parishes. Dolphins that stranded along the coastline of the Florida Panhandle were defined as counties from the Alabama-Florida line to Franklin County. This region was assessed due to the inclusion of the Florida Panhandle as part of the ongoing cetacean UME. The remaining Florida regions were combined (Wakulla to Monroe Counties) and defined as Western Florida.

Within each study region, the monthly total number of dolphin strandings was calculated for each year from January 1990 to June 2013. A baseline for regional monthly average stranding rates was established using a negative binomial log-linear regression model including region, month, the region x month interaction term, and a “coverage” factor. The negative binomial is an appropriate distribution for these count data due to the presence of extra-Poisson variation as demonstrated by significant likelihood ratio tests between Poisson and negative binomial models (data not shown). Each term was included in the regression model as a fixed factor in PROC GLIMMIX (SAS 9.2).

The “coverage” factor was included as a main effect in the model to account for varying degrees of coverage by the MMSN at different times. A historical review of MMSN activity was conducted to identify times and places where coverage was lacking completely (coverage = 0), had limited or only *ad hoc* coverage (e.g. MMSN responder may have recorded cases reported to them but did not examine/recover carcasses (coverage = 1), or had full coverage and a designated active MMSN responder (coverage = 2)). One Florida region (Wakulla through Citrus Counties) has had limited to no MMSN coverage throughout this time series, and Louisiana had little to no MMSN coverage prior to 1993. These periods for these Florida counties and Louisiana were excluded from the analysis of baseline. Limited MMSN coverage occurred during 1998–2007 in Louisiana, September 2005 to June 2007 in Mississippi, and September 2005 to December 2009 in Alabama. The stranding response capability in each of these regions was strongly impacted by Hurricane Katrina in August 2005. The remaining regions and time periods were determined to have “full” MMSN coverage. The inclusion of this factor in the negative binomial regression model reflects the expectation that the number of reported strandings is affected by the presence and activity level of the responsible MMSN member.

Fitted values from the negative binomial regression model provide a mean expectation of the monthly number of dolphin strandings within each region. Observed values that exceeded the upper 95% confidence limit of this prediction were described as “outliers”. The magnitude of monthly numbers of stranded dolphins by region for years 2010, 2011, 2012, and January through June 2013 over or under the 95% confidence limit was calculated (= *actual number of stranded dolphins/95% confidence limit of stranded dolphins*). By doing so, months within regions where stranding numbers could be considered above a defined upper baseline limit from this historical model were identified.

## Results

### Reported stranded dolphins in the GoM, January 1990 through June 2013

A total of 8,149 stranded dolphins were reported in the GoM during 1990 through June 2013. The mean and median annual number of stranded dolphins for the GoM as a whole (1990 to 2012) was 341 (SD = 94) and 318 (range 212–538), respectively. The upper baseline limits (95^th^ quantile) of annually reported stranded dolphins by state (1990 to 2009) were 251 (Texas), 94 (Louisiana), 74 (Mississippi), 53 (Alabama), and 167 (Gulf Florida counties).

### Large scale mortality years (LSMYs) by state, 1990–2012

**Fig 2:**
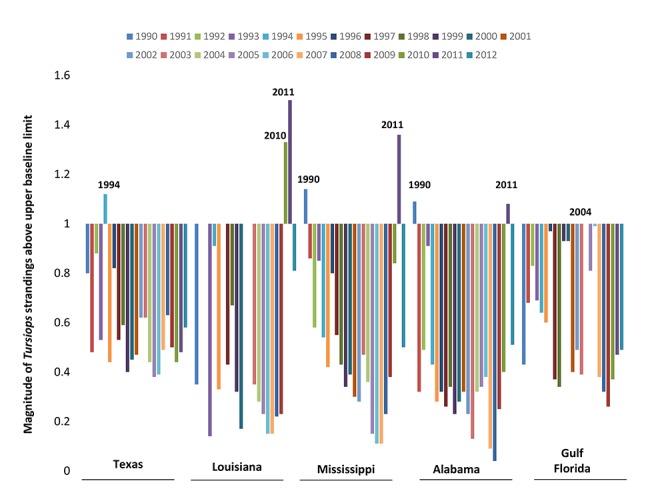
Magnitude of annual common bottlenose dolphin (*Tursiops truncatus*) strandings by state and year above the 95^th^ baseline quantile (1990–2009) in the Gulf of Mexico, 1990 to 2012. State-years with dolphin stranding numbers equal to or greater than ‘1’ are considered large scale mortality years (LSMYs). Louisiana annual stranding data were not included for 1991–1992 and 2001–2002 for Louisiana due to inadequate stranding response coverage during those years.

LSMYs identified before the ongoing UME were 1994 for Texas; 1996 for Louisiana; 1990 for both Mississippi and Alabama; and 2004 for Gulf Florida (Fig. 2). During the ongoing GoM UME (full years 2010–2012), Louisiana had back-to-back LSMYs (2010–2011), and Alabama and Mississippi had one LSMY (2011). Louisiana, Mississippi, and Alabama had total annual, statewide dolphin stranding numbers below the LSMY limit during 2012. No years during the ongoing UME (2010 through 2012) were LSMYs for Gulf Florida or Texas.

Shared LSMYs for Mississippi and Alabama during 1990 and 2011 provided an opportunity to compare the demographics between historical (1990) and the recent GoM UME (2011) strandings. Compared to 1990, stranded dolphins in Mississippi and Alabama during 2011 were approximately 3 times more likely to be perinates, but there were no differences in the proportion of females and males (Table 1). Additionally, LSMYs within the ongoing UME were compared to assess potential demographic clusters. Stranded dolphins in Mississippi and Alabama were also 3 times more likely to be perinates and females compared to dolphins stranded in Louisiana during 2011. Within Louisiana, there was a higher proportion of female *versus* male strandings during 2010 compared to 2011.

**Table 1:** Comparison of demographics for common bottlenose dolphins (*Tursiops truncatus*) stranded in Mississippi and Alabama during shared large-scale mortality years (LSMY) 1990 and 2011.

LSMYs previous to *versus* during the ongoing GoM UME			
**Mississippi**	**1990**	**2011**	***P* value**
Sex (% female)	17/49 (34.7%)	36/77 (46.8%)	0.18
Percent perinates	14/76 (18.4%)	40/96 (41.7%)	< 0.01
Mean body length (cm)	193	149	< 0.01
**Alabama**	**1990**	**2011**	
Sex (% female)	21/49 (42.9%)	21/49 (42.9%)	1.0
Percent perinates	7/52 (13.5%)	30/56 (53.6%)	< 0.01
Mean body length (cm)	201	140	< 0.01
LSMYs during the ongoing GoM UME			
**Louisiana**	**2010**	**2011**	***P* value**
Sex (% female)	37/75 (49.3%)	25/88 (28.4%)	< 0.01
Percent perinates	7/102 (6.9%)	20/134 (14.9%)	0.05
Mean body length (cm)	158	182	0.06
**Louisiana vs. Mississippi & Alabama**	**LA 2011**	**MS & AL 2011**	
Sex (% female)	25/88 (28.4%)	57/126 (45.2%)	0.01
Percent perinates	20/134 (14.9%)	70/152 (46.1%)	< 0.01
Mean body length (cm)	182	146	< 0.01

Louisiana 2011 was compared to Mississippi and Alabama 2011 due to Louisiana’s relative longevity of high stranding rates. The ongoing dolphin Gulf of Mexico (GoM) unusual mortality event (UME) includes years 2010 and 2011.

### Regional baselines

**Fig 3:**
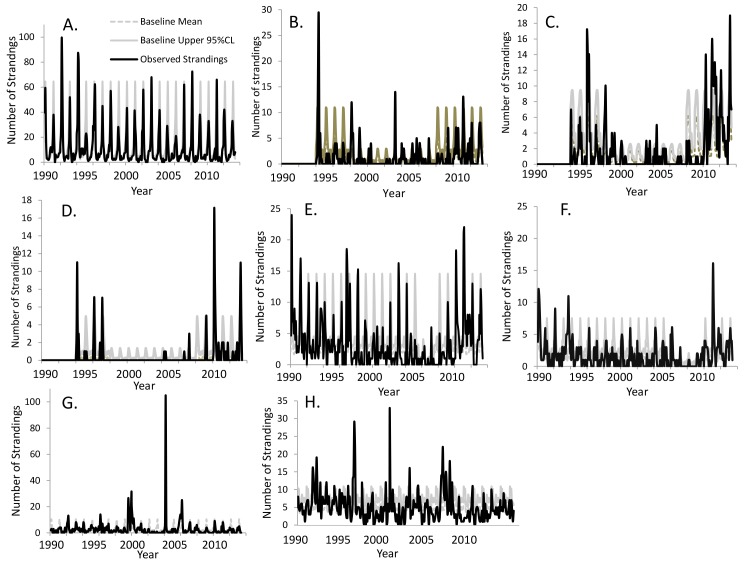
Baseline historical stranding numbers of common bottlenose dolphins (*Tursiops truncatus*) in the northern Gulf of Mexico, fitted values, and the upper 95% confidence limit from negative-binomial regression model for regions with statewide annual increases during the ongoing northern Gulf of Mexico unusual mortality event; A) Texas, B) Western Louisiana, C) Barataria Bay, Louisiana D) Northern Louisiana, E) Mississippi, F) Alabama, G) Florida Panhandle, H) Western Florida.

The negative binomial regression model for all dolphins effectively fit the baseline stranding numbers by month for each region (Fig. 3). Each term in the model (coverage, month, and region) was highly significant (*P* < 0.0001) and provided significant explanatory power [16]. Examination of residual plots indicated that the negative bionomial distribution was appropriate for describing these overdispersed count data. The historical baseline patterns in regions within the boundaries of the northern GoM cetacean UME show strong seasonal patterns in the numbers of strandings, with peak numbers typically occurring in March. The effect of the coverage variable is apparent in both the data and the fitted model, with the number of detected strandings during low coverage periods expected to be 30% of those during the full coverage periods.

### Magnitude of dolphin stranding numbers above baseline during January 2010 to June 2013

**Fig 4:**
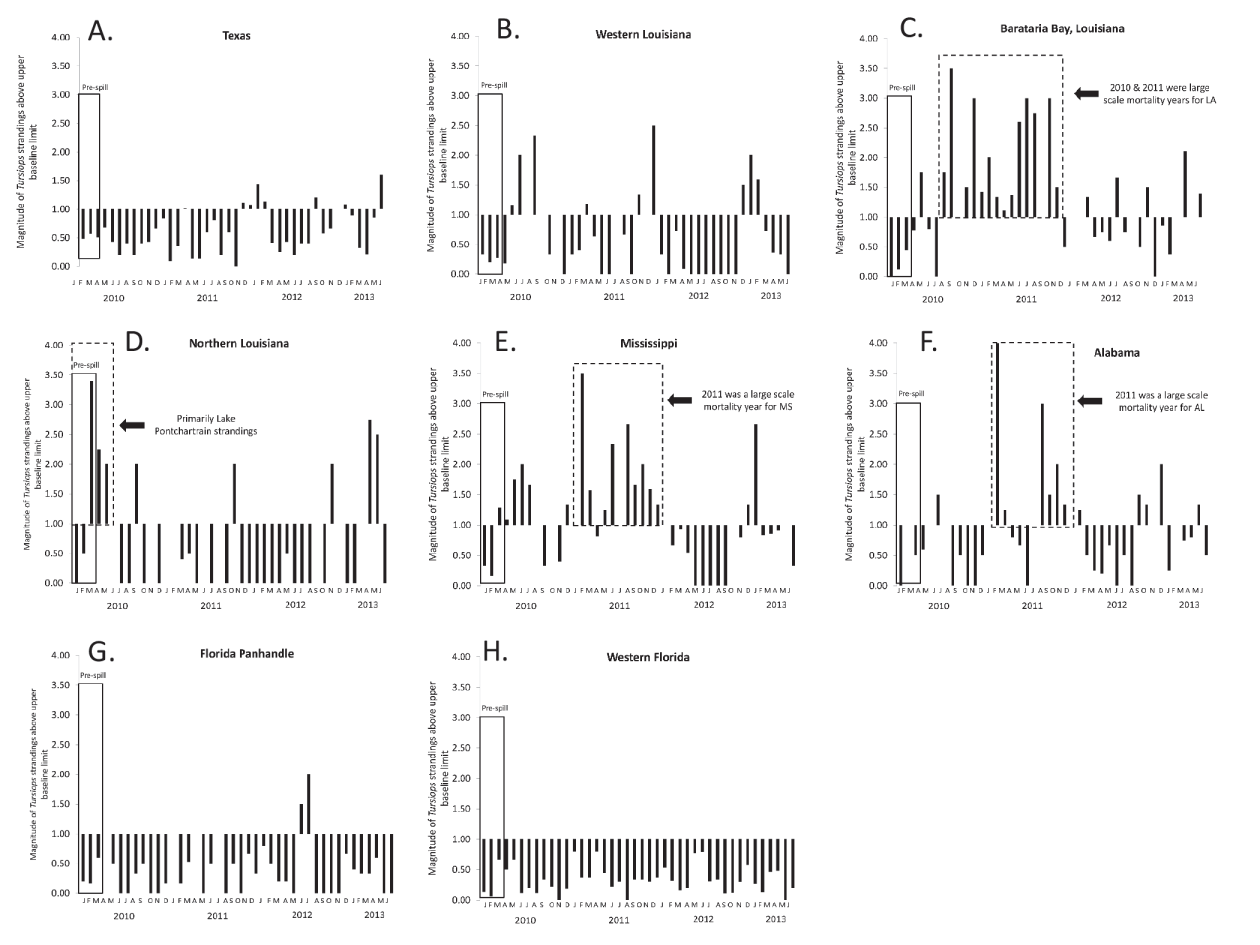
Magnitude of numbers of stranded common bottlenose dolphins (*Tursiops truncatus*) above the upper baseline limit (1990–2009) in the northern Gulf of Mexico during the ongoing unusual mortality event (January 2010-June 2013). From west to east; A) Texas, B) Western Louisiana C) Barataria Bay, Louisiana, D) Northern Louisiana, E) Mississippi, and F) Alabama, G) Florida Panhandle, and H) Western Florida. The solid box marked ‘Pre-spill’ represents time before the *Deepwater Horizon* oil spill in the northern Gulf of Mexico.

The magnitude of stranded dolphins above the upper limit of baseline, by region and month, are provided for 2010 through June 2013 in Fig. 4. High magnitude stranding clusters (multiple, continuous months with stranding numbers above the upper baseline limit) within LSMYs during the GoM UME included clusters during March 2010—May 2010 in northern Louisiana and Mississippi; August 2010—December 2011 in Barataria Bay, Louisiana; and February 2011—December 2011 in Mississippi and Alabama.

Of GoM states that did not have LSMYs during the ongoing GoM UME, Texas had a number of months, particularly late 2011 to early 2012, with elevated numbers of strandings, and the Florida panhandle had elevated strandings during the summer of 2012 (Fig. 4A, G). Elevated strandings in Texas during late 2011 to March 2012 were concurrent with a fish die-off and were declared a separate UME for bottlenose dolphins [17]. The magnitude of Texas strandings above the upper baseline limit was lower than that for Louisiana, Mississippi and Alabama.

Louisiana, Mississippi and Alabama all had months during 2010–2011 in which numbers of stranded dolphins were approximately 3.5 to 4 times above the baseline upper limit. While various monthly stranding totals exceeded the upper baseline limit in GoM states during 2012 through June 2013, increases were not as consistently elevated as during mid-2010 through 2011. Months with elevated numbers of strandings from January 2012 through June 2013 were January, February, and September 2012 and January and June 2013 in Texas; March, July, and November 2012 and April and June 2013 in Barataria Bay, Louisiana; November 2012 and April and May 2013 in northern Louisiana; December 2012 and January 2013 in Mississippi; and January, September, October and December 2012 and May 2013 in Alabama.

## Discussion

The current study identified multiple clusters of bottlenose dolphin strandings with varying spatial and demographic characteristics over the first three and a half years of the ongoing northern GoM UME. During this time, Louisiana had the highest annual dolphin stranding numbers on record for both 2010 and 2011, and the numbers of dolphin mortalities in 2011 were among the highest on record for both Mississippi and Alabama. Identification of multiple spatial, temporal, and demographic clusters within the GoM UME may reflect different etiologies, differences in dolphin population susceptibilities, varying exposures and different susceptibilities to a single etiology, and/or differences in cumulative effects of multiple etiologic factors.

The earliest GoM UME temporal stranding cluster was in northern Louisiana and western Mississippi from March 2010 to May 2010. This cluster included a large number (n = 26) of dolphins that stranded in and around Lake Pontchartrain, Louisiana. These dolphins may have been a part of a distinct group utilizing the lake that NMFS had been monitoring since 2007 [18]. Known drops in temperature and salinity, as well as skin lesions consistent with freshwater exposure, were documented for live Lake Pontchartrain dolphins, suggesting that cold weather and fresh water may have contributed to this stranding cluster [2]. Unfortunately, an investigation into the causes of deaths of Lake Pontchartrain and western Mississippi dolphins in early 2010 has been limited due to advanced decomposition of the recovered carcasses (NMFS, unpublished data).

The longest temporal cluster in the current study involved sustained, higher than usual dolphin stranding numbers in Barataria Bay, Louisiana from August 2010 through December 2011. The timing and location of this cluster is consistent with the spatial and temporal distribution of oil to bay, sound, and estuary habitats in that region during and after the DWH oil spill [7]. Health assessments of live bottlenose dolphins in Barataria Bay conducted during 2011 demonstrated an unexpectedly high prevalence of moderate to severe lung disease and physiologic abnormalities consistent with hypoadrenocorticism [11]. Exposure to DWH oil was proposed as a contributor to these disease states in live Barataria Bay dolphins. Histologic analysis of stranded dolphin carcasses in Barataria Bay and other northern GoM locations, including evaluation of adrenal and lung lesions, is being carried out as part of another study.

This study confirmed both a high prevalence and number of stranded perinatal dolphins in Mississippi and Alabama during early 2011. Combined exposures of pregnant females to unusually cold temperatures, freshwater runoff, and DWH oil have been proposed as the cause of the higher numbers of perinate strandings during 2011 [10]. Additionally, early reports from NMFS suggested *Brucella* infections, which can lead to late-term fetal losses in dolphins, as a possible cause or contributor in these deaths [1,19]. Due to an unknown baseline prevalence of *Brucella* in perinatal dolphins that stranded outside the ongoing UME, however, it is difficult to interpret the significance of these early reported findings. Analysis of tissue lesions and other diagnostics, which was ongoing at the time of this study, will help identify how any one or combination of these factors may have dramatically increased stranding numbers, especially of perinates, in this cluster from Mississippi and Alabama during 2011.

Beyond Barataria Bay, Louisiana, the location, timing, and magnitude of dolphin stranding trends observed following the DWH oil spill, particularly statewide for Louisiana, Mississippi, and Alabama, overlap with the location and magnitude of oil during and the year following spill [7]. In comparison, the GoM coasts of Florida and Texas experienced little to no oiling, and within the UME period through June 2013 these areas lacked significant annual, statewide increases in stranded dolphins [7]. Increased numbers of marine mammal mortalities following oil spills have occurred before; following the *Exxon Valdez* oil spill, increased marine mammal mortalities and/or decreased survival of northern sea otters (*Enhydra lutris kenyoni*), harbor seals (*Phoca vitulina richardsi*), and killer whales (*Orcinus orca*) were reported [20–22]. Thus, the co-occurrence in location and timing of nearshore oil with increased dolphin strandings identified in this study are compelling.

In addition to critical diagnostic testing of stranded dolphins from this UME to assess other potential causes and contributors (e.g. brevetoxicosis and morbillivirus), modeling is needed to better test the associations between dolphin stranding locations and relative areas, magnitude, and duration of oil exposure. In addition to Louisiana, Mississippi, and Alabama, future comparisons should include specific counties in Gulf Florida whose bays, shorelines, and estuaries may have also received DWH oiling, though to a lesser extent.

The current study included stranding data that were available through June of 2013; therefore, interpretations for 2013 are limited. However, high stranding levels were detected in several GoM regions, including Texas, during the first half of 2013. This increase in strandings emphasizes the need to continue to assess demographic trends and potential changes in contributing factors to mortality over time. Closures of UMEs are often based upon return to baseline stranding numbers for a series of months and/or resolution of abnormal pathologic lesions, if identified. Until these criteria are met, the UME may not be eligible for closure. As such, this study demonstrates the importance of both UME declarations, which are based upon independent scientists’ interpretation of data provided to aid effective and immediate response; and LSMYs, a statistical exercise to understand longer-term trends and the relative magnitude of strandings over time.

Increased public awareness, reporting, and marine mammal stranding network response during and following the DWH spill may have influenced the numbers of dolphins detected by responders. If increased reporting was the only factor causing the higher magnitude of stranded dolphins, however, the greatest increase may have been expected immediately following the DWH spill when people were actively combing the coastline during oil cleanup operations and when response vessels were on the water. The formal DWH response period for the northern GoM lasted through November 2, 2010; a portion of Louisiana reopened and reclosed response on December 3, 2010 and May 25, 2011, respectively [23]. Stranding rates were highest in all three states throughout 2011. While some DWH spill cleanup was ongoing at the time of writing this report, the level of activity has been low relative to the initial 2010 response. Therefore, it is unlikely that the increased numbers of strandings observed after the DWH oil spill was driven solely by increased effort and awareness. Future studies may benefit by classifying and comparing time periods with active surveillance to further control for these potential effects.

While the cause(s) of the northern GoM UME remains under investigation, this study revealed that the current UME is composed of multiple clusters of bottlenose dolphin deaths, including some that overlap both temporally and spatially with the DWH oil spill. This information can help to direct the ongoing investigation towards a variety of potential contributing factors over time and geographic locations. Evaluations of lesions and other diagnostic testing of dolphins from the UME will provide critical insight regarding disease processes present and contributors to morbidity and mortality. Beyond aiding with the investigation of this UME, this study demonstrates the importance of sustaining long-term, wildlife health surveillance programs to determine baselines and understand the impacts of changing environments over time.
